# The Effects of Storage Conditions and Homogenisation Buffers on the Measurement of SOD, CAT and ADA Enzyme Activities in Cattle Liver

**DOI:** 10.1002/vms3.70418

**Published:** 2025-05-28

**Authors:** Burcu Menekse Balkan, Ogunc Meral, Bekir Cetintav, Hidayet Tutun, Guzin Ozkurt, Tevhide Sel

**Affiliations:** ^1^ Department of Biochemistry Faculty of Veterinary Medicine Burdur Mehmet Akif Ersoy University Burdur Turkey; ^2^ Department of Biochemistry Faculty of Veterinary Medicine Ankara University Ankara Turkey; ^3^ Department of Biostatistics Faculty of Veterinary Medicine Burdur Mehmet Akif Ersoy University Burdur Turkey; ^4^ Department of Pharmacology and Toxicology Faculty of Veterinary Medicine Burdur Mehmet Akif Ersoy University Burdur Turkey; ^5^ Department of Biochemistry Faculty of Veterinary Medicine Aksaray University Aksaray Turkey

**Keywords:** adenosine deaminase, catalase, cattle, enzymatic activity, liver, superoxide dismutase

## Abstract

**Background:**

Accurate measurement of enzyme activities is very important in studies to evaluate enzymatic parameters. While performing these measurements, many factors can affect the results, including the method of obtaining the tissues, the conditions under which they are stored until analysis, and the methods of determining enzyme activity.

**Objectives:**

This study aimed to investigate the effect of different storage conditions (time and temperature) and different homogenisation buffers (PBS or KCl) on the enzymatic activity of superoxide dismutase (SOD), catalase (CAT), and adenosine deaminase (ADA) in homogenised cattle liver.

**Methods:**

Fresh liver tissue samples were obtained from the slaughterhouse and homogenised in different homogenisation buffers. Supernatants from each sample were divided into three groups according to the experimental design of the study. SOD, CAT and ADA enzyme activities in homogenised tissues were evaluated.

**Results:**

Our data revealed that SOD, CAT and ADA activities did not differ significantly between PBS and KCl groups or between ‐20°C and ‐80°C freezing temperatures. However, our results showed that SOD levels decreased over time in both groups, CAT levels demonstrated a significant decrease from Month 0 to Month 3 and ADA levels decreased significantly over time.

**Conclusions:**

The results indicated that storage time had a significant effect on enzyme activity changes, but the effect of storage temperature and homogenisation buffer was generally limited. These results may support the measurement of enzymatic activity in liver homogenate immediately and, if necessary, after freezing for the shortest possible time.

## Graphical abstract

It is important to accurately measure enzyme activities in research studies to evaluate parameters, and many factors may affect the results of the measurements. The aim of this study was to investigate the effects of storage conditions (time and temperature) and different homogenisation buffers (PBS or KCl) on the measurement of SOD, CAT and ADA enzyme activities in the liver.

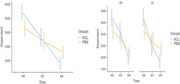

## 1 Introduction

Oxidative stress is caused by the increased production of reactive oxygen species (ROS) such as superoxide (O2^•−^) and hydroxyl radical (OH^•^) due to the inability of the antioxidant system to effectively remove the generated ROS (Feairheller et al. 2011). Lipid peroxidation, which is triggered by ROS, is the most important consequence of oxidative stress and plays an important role in the formation of various diseases due to oxidative damage (Niki 2014). Cellular antioxidant systems are represented by enzymatic and non‐enzymatic antioxidants. The effectiveness of this system depends on the consumption of nutrients such as vitamins and minerals with antioxidant capacity and the production of enzymes such as catalase (CAT) and superoxide dismutase (SOD), which scavenge O2^•−^ radicals and hydrogen peroxide (H_2_O_2_) by converting them into less reactive species. These antioxidant enzymes are also used as markers of oxidative stress (Carmo de Carvalho e Martins et al. 2022; Pandey and Rizvi 2010), and many biomarkers and their measurement methods have been developed to evaluate lipid peroxidation and oxidative stress levels in biological samples (Niki 2014).

SODs are metalloenzymes that catalyse the dismutation of O2^•−^ to H_2_O_2_ and oxygen. H_2_O_2_ is rapidly converted to the highly reactive OH^•^ radical by the Fenton reaction (Pandey and Rizvi 2010). SOD is the front line of defence against ROS‐mediated damage. SOD activity is considered as a biomarker of oxidative stress in patients with several renal, hepatic, and testicular diseases (Pawlak et al. 2005; He et al. 2022; Resim et al. 2015).

CAT is an enzyme that has a very important role in the antioxidant defence system and is responsible for the conversion of H_2_O_2_ into water (H_2_O) and molecular oxygen (O_2_) (Carmo de Carvalho e Martins et al. 2022). The measurement of catalase activity is used as an important and practical biomarker to assess the oxidation‐reduction status and oxidative stress in various biological samples (Carmo de Carvalho e Martins et al. 2022; Stagos et al. 2015).

Adenosine deaminase (ADA) is an enzyme of the purine salvage pathway that catalyses the hydrolytic deamination of adenosine and deoxyadenosine to inosine and deoxyinosine, respectively (Cristalli et al. 2001; Gupta and Nair 2006). It is localised in many tissues, including the lymphoid system, gastrointestinal tract, spleen, skin, brain and kidney. ADA is expressed in almost all mammalian tissues and plays a critical role in the differentiation and maturation of the immune system. ADA is used as a crucial biomarker for assessment of several diseases, such as cancer and pulmonary diseases (Bradford et al. 2017; Beyazit et al. 2012; Flinn and Gennery 2018; Ebrahimi‐Rad et al. 2018).

Many enzyme activities used in monitoring and evaluating various biological processes can be measured with different methods. ADA, CAT and SOD activities can be measured with various methods, including spectrophotometric, colorimetric and high‐performance liquid chromatography (HPLC) in different biological samples such as serum, plasma and tissue homogenates. These enzymes are commonly used in studies on oxidative stress‐related diseases, and the liver appears to be a very effective tissue, along with other tissues, in measuring these enzyme levels. ADA, CAT and SOD activities have been investigated in many diseases, and it has been shown that they can be helpful in the diagnosis and monitoring of the progression of these diseases (Carmo de Carvalho e Martins et al. 2022; Özkurt et al. 2012; Böhmer et al. 2011; Peskin and Winterbourn 2017; Paul et al. 2005).

To the best of our knowledge, it is important to accurately measure enzyme activities in studies to evaluate various parameters, such as oxidative stress, and many factors may affect the results of the measurements, including the method of obtaining the tissues, the conditions in which they are kept until analysis, and the methods of enzyme activity determination. The variability of the storage time, conditions and the homogenisation method may affect the measurement results of enzyme activity, but there is no detailed data examining these parameters according to these variables. Therefore, the aim of this study was to investigate the effects of storage conditions (time and temperature) and different commonly used homogenisation buffers (PBS or KCl) in extraction on the measurement of SOD, CAT and ADA enzyme activities in the liver. The results of this study will enable us to perform comparative analysis of different homogenisation buffers under different storage conditions on the measurement of SOD, CAT and ADA enzyme activities in the liver tissue type for which such comparative data are scarce.

## 2 Materials and Methods

### 2.1 Samples

The liver tissue samples were collected from already slaughtered cattle of the same age and breed as a part of a routine production in the slaughterhouse in Burdur province of Turkey during July 2020. The rapidly harvested samples were quickly transported on ice to the laboratory within an hour of collection and then were washed with 0.9% NaCl and dried with blotting paper.

### 2.2 Experimental Design

The liver tissue samples were homogenised in 1:10 w/v of phosphate‐buffered saline (PBS, pH 7.4) that contains 137 mM NaCl, 2.7 mM KCl, 10 mM Na_2_HPO_4_, 1.8 mM KH_2_PO_4_ or potassium chloride (KCl) buffer that contains 0.15 M KCl on ice by using a homogeniser (Ultra Turrax Type T25‐B, IKA Labortechnic, Germany) for 5 min and centrifuged at 3000 x g for 20 min at 4°C. The supernatants from each homogenisation buffer were divided into three groups. The enzymatic analysis of the first group was performed on the same day the sample arrived in the laboratory. Homogenised samples from the second group were stored in a temperature‐controlled incubator set at ‐20°C while the samples from the third group were stored at ‐80°C. Some of the homogenised samples in the second and third groups were subjected to enzymatic analysis after 3 months under different storage conditions (‐20 or ‐80°C). Enzymatic analysis of the remaining samples was performed at the end of the sixth month.

### 2.3 Determination of Superoxide Dismutase Activity

SOD activity of homogenised tissue samples was measured by the method described by Sun et al. (1988). In the method of SOD activity, superoxide, which is produced by an enzymatic reaction of xanthine oxidase, causes nitroblue tetrazolium (NBT) reduction in the sample, and this reaction is inhibited by SOD. Superoxide radicals react with NBT and results formation of formazone, which gives maximum absorbance at 560 nm. The enzyme slows down the NBT reduction reaction and, as a result, causes a decrease in the absorbance values in the spectrophotometer. SOD activity was measured at 560 nm on a spectrophotometer by the degree of inhibition of the reaction of homogenised tissue. Intra‐assay and inter‐assay coefficient variation (CV%) of SOD analysis were <3% and <4%, respectively. The enzyme activity was expressed as U/g protein. Each sample was measured twice.

### 2.4 Determination of Catalase Activity

Catalase (CAT) activity of samples was measured according to the method reported by Aebi (1983). Briefly, test and blank tubes were prepared in the experiment. Phosphate buffer and samples were placed in a blank tube, and samples prepared in phosphate buffer and H_2_O_2_ were placed in a sample tube. Absorbance values were measured at 240 nm. The time required for the absorbance to decrease from 0.45 to 0.40 was recorded. Intra‐assay and inter‐assay coefficient variation (CV%) of CAT analysis were <4% and <7% respectively. The result was calculated as k. k = (0.1175 / Δt) x sn^−1^ and enzyme activity was expressed as k/g protein. Each sample was measured twice.

### 2.5 Determination of Adenosine Deaminase Activity

ADA enzyme activity in the supernatants was measured by the method described by Guisti (1974). Briefly, sample, blank, standard and standard blank tubes were prepared. Adenosine solution and samples were added to the sample tube; adenosine solution was added to the sample blank tube; standard solutions and distilled water were added to the standard tube; phosphate buffer and distilled water were added to the standard blank tube; and all tubes were incubated in a 37°C water bath for 60 min. Phenol nitroproside was added to all tubes following incubation. Then, alkaline hypochlorite solution was added to all tubes after adding the sample to the standard blank tube. After mixing, test tubes were incubated in a 37°C water bath for another 30 min. The absorbance (OD) in the sample and blank test tubes was read against distilled water at a wavelength of 628 nm. Intra‐assay and inter‐assay coefficient variation (CV%) of ADA analysis were <3% and <6%, respectively. Each sample was measured twice. The ADA activity (U/g protein) was calculated as;

$$\left[\left(\mathrm{SOD}-\mathrm{SBOD}\right)/\left(\mathrm{StOD}-\mathrm{StBOD}\right)\right]\times F$$

F: Factor value determined by the study, which is the amount of ammonia produced in 1 min.
SOD: Sample optical density
SBOD: Sample blank optical density
StOD: Standard optical density
StBOD: Standard blank optical density

### 2.6 Determination of Protein Concentration

The Bradford assay (Bradford 1976) was used to estimate the total protein concentration in the supernatants.

### 2.7 Statistical Analysis

Repeated measures ANOVAs were conducted to assess the effects of two fixed factors—grouping variable (PBS vs. KCl) and freezing temperature (‐20°C vs. ‐80°C)—on biochemical parameters (SOD, CAT, ADA). The study design included two levels of the grouping variable (PBS and KCl) and freezing temperature conditions (‐20°C and ‐80°C). Enzyme activities were measured at three time points (0, 3, and 6 months). The most significant time points were selected to examine changes in enzyme activity. The sample size of animal material was decided using G*Power software (version 3.1.9.7).

The assumption of normality was verified using the Shapiro‐Wilk test, which indicated no significant deviations from normality across all groups. Additionally, the assumption of sphericity was tested using Mauchly's test, which was non‐significant for each measurement. Consequently, no corrections for sphericity were applied. Post hoc comparisons were performed using the Tukey correction. All analyses were conducted using JAMOVI (version 2.3.2), and significance was set at p < 0.05.

## 3 Results

### 3.1 Effects of Storage Conditions and Homogenisation Buffers on SOD Enzyme Activity

**FIGURE 1:**
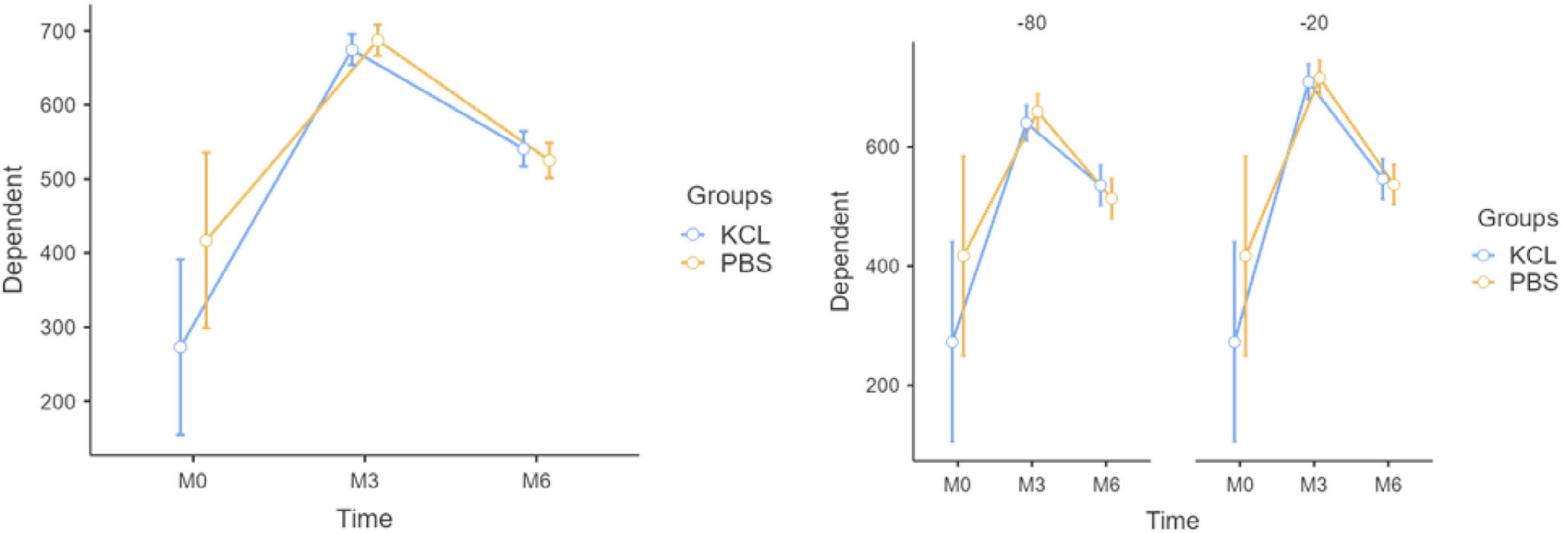
Effects of storage conditions and homogenisation buffers on SOD enzyme activity.

The SOD enzyme analysis indicated no significant main effects of the fixed factors (grouping variable or freezing temperature) on SOD levels (p > 0.05). This implies that SOD levels did not vary significantly between PBS and KCl groups or between the freezing temperatures of ‐20°C and ‐80°C. However, a significant effect of the repeated measures factor (Time) was observed (F = 45.5118, p < 0.001), indicating that SOD levels changed significantly over time. In contrast, the interaction between time and group was not statistically significant (F = 2.9203, p = 0.060), suggesting that the changes in SOD levels over time were consistent across the PBS and KCl groups (Table 1). As illustrated in Figure 1, SOD levels decreased over time in both groups, but no substantial differences were observed between the groups' trajectories.

**TABLE 1:** Comparison of SOD (U/g protein) activity between groups.

Estimated marginal means—Time ★ Buffer ★ Temperature							
						95% Confidence interval	
Temperature	Buffer	Time	n	Mean	SE	Lower	Upper
−80°C	KCl	Month 0	11	273	82.9	105	440
		Month 3	11	640	14.5	611	669
		Month 6	11	536	16.5	502	569
	PBS	Month 0	11	417	82.9	250	584
		Month 3	11	659	14.5	630	688
		Month 6	11	513	16.5	480	547
−20°C	KCl	Month 0	11	273	82.9	105	440
		Month 3	11	709	14.5	680	739
		Month 6	11	546	16.5	513	579
	PBS	Month 0	11	417	82.9	250	584
		Month 3	11	715	14.5	686	745
		Month 6	11	537	16.5	503	570

### 3.2 Effects of Storage Conditions and Homogenisation Buffers on CAT Enzyme Activity

**FIGURE 2:**
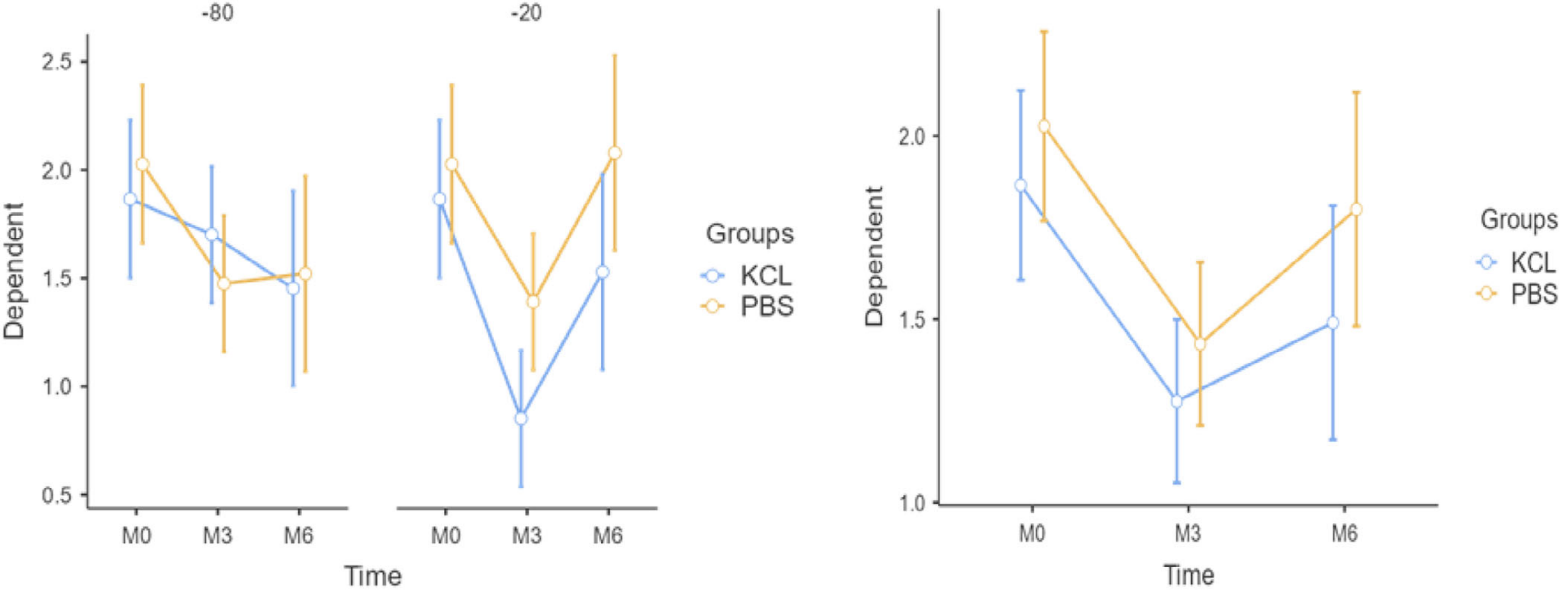
Effects of storage conditions and homogenisation buffers on CAT enzyme activity.

The CAT enzyme analysis indicated no significant main effects of the fixed factors (grouping variable or freezing temperature) on CAT levels (**p > 0.05**). This implies that CAT levels did not vary significantly between PBS and KCl groups or between the freezing temperatures of ‐20°C and ‐80°C. However, a significant effect of the repeated measures factor (Time) was observed (**F = 11.547, p < 0.001**), indicating that CAT levels changed significantly over time (Table 2). Additionally, a significant interaction between time and temperature groups was found (**F = 5.126, p = 0.008**), suggesting that the change in CAT levels over time varied between the freezing temperature conditions (‐20°C and ‐80°C). In contrast, the interaction between time and group was not statistically significant (**F = 0.249, p = 0.780**), indicating consistent changes over time across PBS and KCl groups. The three‐way interaction (Time ★ Groups ★ Temp. Groups) was also not significant (**p = 0.296**). As illustrated in Figure 2, CAT levels demonstrated a significant decrease from Month 0 to Month 3 (**p < 0.001**), while the change from Month 3 to Month 6 was not statistically significant (**p = 0.071**). These results highlight the importance of considering freezing temperature when interpreting temporal changes in CAT levels.

**TABLE 2:** Comparison of CAT (k/g protein) activity between groups.

Estimated marginal means—Time ★ Buffer ★ Temperature							
						95% Confidence interval	
Temperature	Buffer	Time	n	Mean	SE	Lower	Upper
−80°C	KCl	Month 0	11	1,86	0,18	1,50	2,23
		Month 3	11	1,70	0,15	1,38	2,02
		Month 6	11	1,45	0,22	1,00	1,90
	PBS	Month 0	11	2,02	0,18	1,66	2,39
		Month 3	11	1,47	0,15	1,16	1,79
		Month 6	11	1,52	0,22	1,07	1,97
−20°C	KCl	Month 0	11	1,86	0,18	1,50	2,23
		Month 3	11	0,85	0,15	0,53	1,17
		Month 6	11	1,52	0,22	1,07	1,98
	PBS	Month 0	11	2,02	0,18	1,66	2,39
		Month 3	11	1,39	0,15	1,07	1,71
		Month 6	11	2,07	0,22	1,62	2,53

### 3.3 Effects of Storage Conditions and Homogenisation Buffers on ADA Enzyme Activity

**FIGURE 3:**
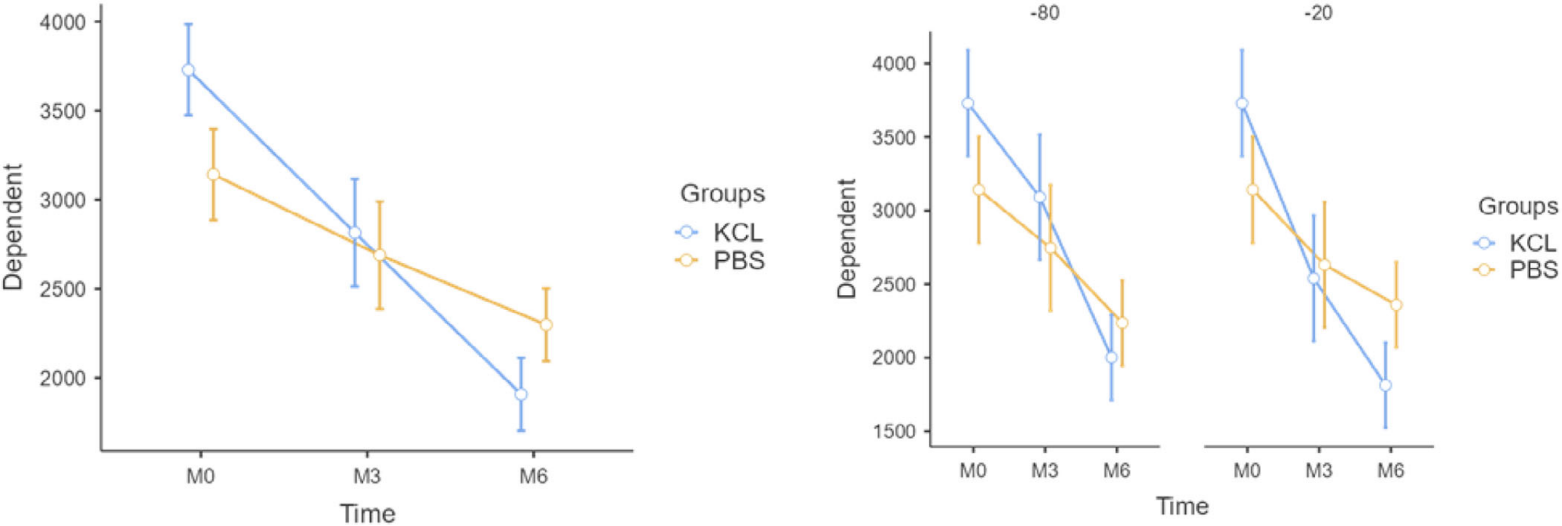
Effects of storage conditions and homogenisation buffers on ADA enzyme activity.

The ADA enzyme analysis indicated no significant main effects of the fixed factors (grouping variable or freezing temperature) on ADA levels (p > 0.05). This implies that ADA levels did not vary significantly between PBS and KCl groups or between the freezing temperatures of ‐20°C and ‐80°C. However, a significant effect of the repeated measures factor (Time) was observed (F = 80.850, p < 0.001), indicating that ADA levels decreased significantly over time (Table 3). Moreover, the interaction between Time and Group was statistically significant (F = 10.897, p < 0.001). As illustrated in Figure 3, the ADA levels in the KCl group showed a more pronounced decrease over time compared to the PBS group.

**TABLE 3:** Comparison of ADA (U/g protein) activity between groups.

Estimated marginal means—Time ★ Buffer ★ Temperature							
						95% Confidence interval	
Temperature	Buffer	Time	n	Mean	SE	Lower	Upper
−80°C	KCl	Month 0	11	3729	179	3368	4090
		Month 3	11	3091	211	2664	3517
		Month 6	11	2002	143	1712	2291
	PBS	Month 0	11	3729	179	3368	4090
		Month 3	11	2539	211	2113	2965
		Month 6	11	1813	143	1523	2102
−20°C	KCl	Month 0	11	3141	179	2780	3502
		Month 3	11	2746	211	2320	3173
		Month 6	11	2236	143	1946	2526
	PBS	Month 0	11	3141	179	2780	3502
		Month 3	11	2631	211	2204	3057
		Month 6	11	2359	143	2069	2649

## 4 Discussion

In enzyme studies, tissue homogenisation methods and repeated freeze‐thaw cycles can cause inaccurate results in enzyme activity measurements. Besides, in cases where enzyme activity measurements need to be performed at a later date, choosing buffer systems that support long‐term enzyme stability and storage of the homogenate under appropriate conditions are crucial for the accuracy of the measurement. In the current study, we investigated the effects of storage conditions on SOD, CAT and ADA enzyme activities in homogenised tissues prepared with different buffers (PBS and KCl). Storage conditions were carried out at two temperatures (‐20 and ‐80°C) and three different periods (months 0, 3 and 6).

Some antioxidant enzymes are sensitive to freeze‐thaw stress, which can alter the enzyme activity. It was observed that catalase was the most resistant antioxidant enzyme to freeze‐thaw stress, while SOD enzyme was resistant to 4 freeze‐thaw stress cycles (Murias et al. 2005). When homogenised liver samples were stored at ‐20°C for 60 days (despite 3 times freeze‐thaw stress), it was observed that CAT and SOD enzyme activities did not change except for a very small decrease on day 30 (Bortolin et al. 2017). In our study, the samples of which enzyme activities were measured at 3 and 6 months were subjected to the freeze‐thaw process once. However, there were significant time‐dependent changes in enzyme activity.

The effect of the storage conditions of the solutions on the enzyme activities can vary depending on the type of buffer and freeze‐thaw protocol. Freezing may lead to structural changes, aggregation and loss of activity in enzymes. It is also known that the freezing rate has an effect on the enzyme activity in solutions (Park et al. 2021). Similarly, the dissolution rate may have a negative impact on enzyme activity (Cao et al. 2003). In our study, although the thawing conditions were similar in both storage conditions, since freezing was performed at two different temperatures, it can be expected that enzyme activities may differ due to the difference in freezing rate (Bao et al. 2023). The similarity in enzyme activity between the groups suggested that the freezing rate difference between ‐20 and ‐80°C may not be strong enough to affect enzyme activity. In contrast to our results, another study reported that the stability of antioxidant enzymes in tissue homogenate was more stable at ‐70°C than at ‐20°C (Jung et al. 1993). In our study, CAT and SOD enzyme activities changed over time, but only CAT activity change differed depending on temperature. This may be due to the effect of different temperatures on protein structure or solubility over time.

Precipitation of buffer components at low temperature (salt crystallisation) causes a pH decrease, which can lead to aggregation of enzymes and loss of enzymatic activity (Cao et al. 2003; Thorat et al. 2020). In our study, the type of homogenisation buffer and freezing temperature did not affect the activity of antioxidant enzymes. It can be concluded that the precipitation of buffer components at low temperatures does not change the enzyme activity of both the homogenisation buffer type and different freezing temperatures.

Freeze‐thaw stress increases oxidative stress due to ROS formation; besides homogenised tissues are more susceptible to oxidative damage (Bortolin et al. 2017; Park et al. 1998; Len et al. 2019). To the best of our knowledge, the amount of ROS in homogenised tissue may increase as the cold storage time increases, which may explain the time‐dependent change in CAT enzyme activity that occurred in our study. Protein denaturation and aggregation, pH change and buffer properties may also have contributed to this change.

In our study, ADA enzyme activity did not change depending on the type of buffer used and the temperature at which the samples were stored (p > 0.05). However, ADA activity decreased with increasing storage time. A similar effect was observed in another study, such as decreased ADA activity occurring in bovine thymus exposed to different freezing conditions (Hajduk 1999). Another study reported that storage of erythrocytes at room temperature or ‐20°C led to a loss of adenosine deaminase activity (Körber et al. 1975). In contrast to these studies, no significant changes in ADA activity were reported in samples stored at different temperatures (+4°C and ‐20°C) for 28 days (Antonangelo et al. 2006). However, in another study it was reported that ADA activity remained stable at ‐80°C for up to 2.6 years, but activity started to decrease with longer‐term storage of pleural fluid samples (Bielsa et al. 2014). In the current study, it was also determined that the decrease in ADA enzyme activity over time was not the same in the PBS and KCl groups. The decrease in ADA enzyme activity over time was found to be slower in liver homogenised with PBS compared to KCl at all temperature conditions. These results showed that ADA activity decreased in both buffers, but PBS buffer maintained ADA enzyme stability better.

In conclusion, our present study demonstrated that storage time had a significant effect on enzyme activity changes, but the effect of storage temperature and homogenisation buffer was generally limited. The results of our study indicate that CAT and ADA enzyme activities were decreased significantly over time. The results of the study may suggest that enzyme activities of homogenised liver tissue should be measured as much as possible before storage, and if storage is necessary, it should be stored at ‐80 C for a short time. A limitation of this study is that the single tissue type (liver) was studied; therefore, the effects of storage conditions and homogenisation buffers on the measurement of SOD, CAT and ADA enzyme activities need to be investigated in future studies, preferably on different tissues.

## Author Contributions

**Burcu Menekse Balkan**: conceptualisation, methodology, investigation, formal analysis, visualisation, writing and review ‐ editing. **Ogunc Meral**: conceptualisation, methodology, investigation, formal analysis, writing and review ‐ editing. **Bekir Cetintav**: methodology, data curation, investigation, formal analysis, visualisation and review ‐ editing. **Hidayet Tutun**: methodology, investigation, formal analysis, writing and review ‐ editing. **Guzin Ozkurt**: methodology, investigation, formal analysis, writing and review ‐ editing. **Tevhide Sel**: conceptualisation, methodology, supervision, investigation and review ‐ editing.

## Ethics Statement

The authors confirm that the ethical policies of the journal, as noted on the journal's author guidelines page, have been adhered to and the study was conducted in accordance with the Directives of the Local Ethic Committee for Animal Experiments of Ankara University (Article No: 8.8.k.2).

## Conflicts of Interest

The authors declare no conflicts of interest.

## Data Availability

The data supporting the findings of this study are available from the corresponding author upon request.

## References

[vms370418-bib-0001] Aebi, H. E. 1983. “Catalase in: H.U.Bermeyer (Hrsy).” In Methods of Enzymatic Analysis, 273–286. Verlag Chemie.

[vms370418-bib-0002] Antonangelo, L. , F. S. Vargas , L. P. Almeida , et al. 2006. “Influence of Storage Time and Temperature on Pleural Fluid Adenosine Deaminase Determination.” Respirology (Carlton, Vic.) 11, no. 4: 488–492. 10.1111/j.1440-1843.2006.00866.x. 16771922

[vms370418-bib-0003] Bao, Y. , Y. Zhang , and W. Xu . 2023. “Effects of Different Freezing Rate and Frozen Storage Temperature on the Quality of Large‐Mouth Bass (Micropterus salmoides).” Molecules (Basel, Switzerland) 28, no. 14: 5432. 10.3390/molecules28145432. 37513304 PMC10385098

[vms370418-bib-0004] Beyazit, Y. , S. Koklu , A. Tas , et al. 2012. “Serum Adenosine Deaminase Activity as a Predictor of Disease Severity in Ulcerative Colitis.” Journal of Crohn's & Colitis 6, no. 1: 102–107. 10.1016/j.crohns.2011.07.010. 22261534

[vms370418-bib-0005] Bielsa, S. , A. Esquerda , R. M. Palma , A. Criado , and J. M. Porcel . 2014. “Influence of Storage Time on Pleural Fluid Adenosine Deaminase Activity.” Clinical Laboratory 60, no. 3: 501–504. 10.7754/clin.lab.2013.130311. 24697129

[vms370418-bib-0006] Böhmer, A. , J. Jordan , and D. Tsikas . 2011. “High‐Performance Liquid Chromatography Ultraviolet Assay for human Erythrocytic Catalase Activity by Measuring Glutathione as o‐Phthalaldehyde Derivative.” Analytical Biochemistry 410, no. 2: 296–303. 10.1016/j.ab.2010.11.026. 21094119

[vms370418-bib-0007] Bortolin, R. C. , J. Gasparotto , A. R. Vargas , et al. 2017. “Effects of Freeze‐Thaw and Storage on Enzymatic Activities, Protein Oxidative Damage, and Immunocontent of the Blood, Liver, and Brain of Rats.” Biopreservation and Biobanking 15, no. 3: 182–190. 10.1089/bio.2016.0023. 27662116

[vms370418-bib-0008] Bradford, K. L. , F. A. Moretti , D. A. Carbonaro‐Sarracino , H. B. Gaspar , and D. B. Kohn . 2017. “Adenosine Deaminase (ADA)‐Deficient Severe Combined Immune Deficiency (SCID): Molecular Pathogenesis and Clinical Manifestations.” Journal of Clinical Immunology 37, no. 7: 626–637. 10.1007/s10875-017-0433-3. 28842866

[vms370418-bib-0009] Bradford, M. M. 1976. “A Rapid and Sensitive Method for the Quantitation of Microgram Quantities of Protein Utilizing the Principle of Protein‐Dye Binding.” Analytical Biochemistry 72: 248–254. 10.1016/0003-2697(76)90527-3. 942051

[vms370418-bib-0010] Cao, E. , Y. Chen , Z. Cui , and P. R. Foster . 2003. “Effect of Freezing and Thawing Rates on Denaturation of Proteins in Aqueous Solutions.” Biotechnology and Bioengineering 82, no. 6: 684–690. 10.1002/bit.10612. 12673768

[vms370418-bib-0011] Carmo de Carvalho e Martins, M.d. , A. S. da Silva Santos Oliveira , L. A. A. da Silva , M. G. S. Primo , and V. B. de Carvalho Lira . 2022. “Biological Indicators of Oxidative Stress [Malondialdehyde, Catalase, Glutathione Peroxidase, and Superoxide Dismutase] and Their Application in Nutrition.” In Biomarkers in Nutrition . Biomarkers in Disease: Methods, Discoveries and Applications, edited by V. B. Patel and V. R. Preedy , Springer. 10.1007/978-3-031-07389-2_49.

[vms370418-bib-0012] Cristalli, G. , S. Costanzi , C. Lambertucci , et al. 2001. “Adenosine Deaminase: Functional Implications and Different Classes of Inhibitors.” Medicinal Research Reviews 21, no. 2: 105–128. 10.1002/1098-1128(200103)21:2<105::aid-med1002>3.0.co;2-u. 11223861

[vms370418-bib-0013] Ebrahimi‐Rad, M. , S. Khatami , S. Ansari , S. Jalylfar , S. Valadbeigi , and R. Saghiri . 2018. “Adenosine Deaminase 1 as a Biomarker for Diagnosis and Monitoring of Patients With Acute Lymphoblastic Leukemia.” Journal of Medical Biochemistry 37, no. 2: 128–133. 10.1515/jomb-2017-0042. 30581348 PMC6294093

[vms370418-bib-0014] Feairheller, D. L. , J. Y. Park , K. M. Sturgeon , et al. 2011. “Racial Differences in Oxidative Stress and Inflammation: in Vitro and in Vivo.” Clinical and Translational Science 4, no. 1: 32–37. 10.1111/j.1752-8062.2011.00264.x. 21348953 PMC3077905

[vms370418-bib-0015] Flinn, A. M. , and A. R. Gennery . 2018. “Adenosine Deaminase Deficiency: A Review.” Orphanet Journal of Rare Diseases 13, no. 1: 65. 10.1186/s13023-018-0807-5. 29690908 PMC5916829

[vms370418-bib-0016] Giusti, G. 1974. “Adenosine Deaminase.” In Methods of Enzymatic Analysis, edited by H. U. Bergmeyer , 2nd ed. 1092–1099. Academic Press.

[vms370418-bib-0017] Gupta, M. , and V. Nair . 2006. “Adenosine Deaminase in Nucleoside Synthesis. A Review.” Collection of Czechoslovak Chemical Communications 71, no. 6: 769–787. 10.1135/cccc20060912 PMC8608517 34815583

[vms370418-bib-0018] Hajduk, E. 1999. “Changes in Some Enzyme Activities and DNA Content in Frozen Stored and Freeze‐Dried Bovine Thymus.” Food Chemistry 66, no. 2: 235–239. 10.1016/S0308-8146(99)00056-4.

[vms370418-bib-0019] He, Y. , F. Wang , N. Yao , Y. Wu , Y. Zhao , and Z. Tian . 2022. “Serum Superoxide Dismutase Level is a Potential Biomarker of Disease Prognosis in Patients With HEV‐Induced Liver Failure.” BMC Gastroenterology 22, no. 1: 14. 10.1186/s12876-022-02095-2. 35000581 PMC8742945

[vms370418-bib-0020] Jung, K. , S. Kühler , S. Klotzek , S. Becker , and W. Henke . 1993. “Effect of Storage Temperature on the Activity of Superoxide Dismutase, Catalase, Glutathione Peroxidase, Glutathione Reductase and Glutathione S‐Transferase in Rat Liver and Kidney Homogenates.” Enzyme & Protein 47, no. 3: 149–155. 10.1159/000468670. 8087206

[vms370418-bib-0021] Körber, W. , E. B. Meisterernst , and G. Hermann . 1975. “Quantitative Measurement of Adenosine Deaminase From Human Erythrocytes.” Clinica Chimica Acta; International Journal of Clinical Chemistry 63, no. 3: 323–333. 10.1016/0009-8981(75)90054-6. 240521

[vms370418-bib-0022] Len, J. S. , W. S. D. Koh , and S. X. Tan . 2019. “The Roles of Reactive Oxygen Species and Antioxidants in Cryopreservation.” Bioscience Reports 39, no. 8: BSR20191601. 10.1042/BSR20191601. 31371631 PMC6712439

[vms370418-bib-0023] Murias, M. , M. Rachtan , and J. Jodynis‐Liebert . 2005. “Effect of Multiple Freeze‐Thaw Cycles of Cytoplasm Samples on the Activity of Antioxidant Enzymes.” Journal of Pharmacological and Toxicological Methods 52, no. 2: 302–305. 10.1016/j.vascn.2005.03.002. 16125630

[vms370418-bib-0024] Niki, E. 2014. “Biomarkers of Lipid Peroxidation in Clinical Material.” Biochimica Et Biophysica Acta 1840, no. 2: 809–817. 10.1016/j.bbagen.2013.03.020. 23541987

[vms370418-bib-0025] Özkurt, G. , A. Gökçen , İ. Çamkerten , T. Şahin , B. M. Balkan , and M. Boz . 2012. “Doğal Enfekte Nematotlu Kilis Keçilerinde Eritrosit SOD, CAT, GPx Enzim Aktiviteleri Ve MDA Düzeyi.” Harran Üniversitesi Veteriner Fakültesi Dergisi 1, no. 2: 107–110.

[vms370418-bib-0026] Pandey, K. B. , and S. I. Rizvi . 2010. “Markers of Oxidative Stress in Erythrocytes and Plasma During Aging in Humans.” Oxidative Medicine and Cellular Longevity 3, no. 1: 2–12. 10.4161/oxim.3.1.10476. 20716923 PMC2835884

[vms370418-bib-0027] Park, H. , J. Y. Park , K. M. Park , and P. S. Chang . 2021. “Effects of Freezing Rate on Structural Changes in L‐Lactate Dehydrogenase During the Freezing Process.” Scientific Reports 11, no. 1: 13643. 10.1038/s41598-021-93127-6. 34211044 PMC8249661

[vms370418-bib-0028] Park, J. I. , C. M. Grant , M. J. Davies , and I. W. Dawes . 1998. “The Cytoplasmic Cu,Zn Superoxide Dismutase of Saccharomyces Cerevisiae is Required for Resistance to Freeze‐Thaw Stress. Generation of Free Radicals During Freezing and Thawing.” The Journal of Biological Chemistry 273, no. 36: 22921–22928. 10.1074/jbc.273.36.22921. 9722512

[vms370418-bib-0029] Paul, M. K. , V. Grover , and A. K. Mukhopadhyay . 2005. “Merits of HPLC‐Based Method Over Spectrophotometric Method for Assessing the Kinetics and Inhibition of Mammalian Adenosine Deaminase.” Journal of Chromatography. B, Analytical Technologies in the Biomedical and Life Sciences 822, no. 1‐2: 146–153. 15993664 10.1016/j.jchromb.2005.06.006

[vms370418-bib-0030] Pawlak, K. , D. Pawlak , and M. Mysliwiec . 2005. “Cu/Zn Superoxide Dismutase Plasma Levels as a New Useful Clinical Biomarker of Oxidative Stress in Patients With End‐Stage Renal Disease.” Clinical Biochemistry 38, no. 8: 700–705. 10.1016/j.clinbiochem.2005.02.009. 15963971

[vms370418-bib-0031] Peskin, A. V. , and C. C. Winterbourn . 2017. “Assay of Superoxide Dismutase Activity in a Plate Assay Using WST‐1.” Free Radical Biology & Medicine 103: 188–191. 10.1016/j.freeradbiomed.2016.12.033. 28017897

[vms370418-bib-0032] Resim, S. , E. B. Kurutas , A. B. Gul , et al. 2015. “The Levels of Oxidative Stress Biomarkers in Rats as a Response to Different Techniques of Testicular Biopsy.” The Indian Journal of Surgery 77, no. Suppl 2: 310–313. 10.1007/s12262-013-0808-5. 26730016 PMC4692931

[vms370418-bib-0033] Stagos, D. , N. Goutzourelas , D. Bar‐Or , et al. 2015. “Application of a New Oxidation‐Reduction Potential Assessment Method in Strenuous Exercise‐Induced Oxidative Stress.” Redox Report: Communications in Free Radical Research 20, no. 4: 154–162. 25494543 10.1179/1351000214Y.0000000118 PMC6837711

[vms370418-bib-0034] Sun, Y. , L. W. Oberley , and Y. Li . 1988. “A Simple Method for Clinical Assay of Superoxide Dismutase.” Clinical Chemistry 34, no. 3: 497–500. 3349599

[vms370418-bib-0035] Thorat, A. A. , B. Munjal , T. W. Geders , and R. Suryanarayanan . 2020. “Freezing‐Induced Protein Aggregation—Role of pH Shift and Potential Mitigation Strategies.” Journal of Controlled Release: Official Journal of the Controlled Release Society 323: 591–599. 10.1016/j.jconrel.2020.04.033. 32335158

